# 

**Published:** 1886-07

**Authors:** 


					﻿V(/l VII. ] TWO DOLLARS/YEAR. SINGLE COPIES 60 CENTS. [No. 3.
THE JOURNAL
OF
Comparative Medicine
and Surgery.
EDITED BY
W. A. CONKLIN, Ph.D., F. S. BILLINGS, D.V.S.
ASSISTED BY
Prof. ALBERT JOHNE, Royal Veterinary School, Dresden, Germany.
THOMAS B. ROGERS, D.V.Sr, Veterinary Department, University of Penn’a.
W. CLEMENT, V.S.j Montreal Veterinary College.
A. T. YOKURA, V.S., Imperial Agricutural School, Japan.
Entered New York Post Office as second class matter.
JULY, 1886
WILLIAM R. JENKINS,
Veterinary Publisher, Bookseller and Printer,
8.50 Sixth Ave., Qor. 48th Street,
NEW YORK.
LONDON; BAILLIERE, TINDALL & COX.
				

